# Oral Myiasis Caused by *Chrysomya bezziana* in Anterior Maxilla

**DOI:** 10.1155/2014/518427

**Published:** 2014-04-29

**Authors:** Ankur Aggarwal, M. Jonathan Daniel, Raju Singam Shetty, Boddu Naresh Kumar, C. H. Sumalatha, E. Srikanth, Shalu Rai, Rohit Malik

**Affiliations:** ^1^Department of Oral Medicine & Radiology, IDST, Modinagar, Ghaziabad, Uttar Pradesh 201201, India; ^2^Department of Oral Medicine & Radiology, Mahatma Gandhi Postgraduate Institute of Dental Sciences, Pondicherry 605006, India; ^3^Department of Orthodontics, Awadh Dental College, Jamshedpur, Jharkhand 831012, India; ^4^Department of Orthodontics, Government Dental College, Hyderabad, Andhra Pradesh 500012, India; ^5^Department of Endodontics, Sibar Institute of Dental Sciences, Guntur, Andhra Pradesh 522509, India

## Abstract

Oral myiasis is a rare pathology and is associated with poor oral hygiene, alcoholism, senility, suppurating lesions, and severe halitosis. It arises from invasion of body tissues or cavities of living animals by maggots or larvae of certain dipterian flies. It is mostly reported in developing countries and in the tropics. We hereby report a rare case of oral myiasis in a 70-year-old female with extensive necrotic oral lesion burrowing into the hard palate through which numerous live maggots (larvae) and seen emerging out and discuss the definition, etiology, predisposing factors, classification, and management of the same. Furthermore, the life cycle of the causative organism in the present case, that is, *Chrysomya bezziana*, has also been discussed.

## 1. Introduction

Myiasis is a term derived from the Greek word “myia,” meaning invasion of vital tissue of humans or other mammals by fly larvae [1]. The term “myiasis” was coined by F. W. Hope in 1840 to refer to diseases of human originating specifically with dipterous larvae, as opposed to those caused by insect larvae in general, scholechiasis [2]. Oral myiasis was first described by Laurence in 1909 [3].

Myiasis is caused by dipterous larvae that feed on the host dead or living tissues, liquid body substances, or ingested food. Myiasis frequently occurs in rural areas, infecting livestock, and in humans prevails in unhealthy individuals in third world countries [4]. Human myiasis is reported mainly in developing countries such as Asian countries and very rarely in western countries [2].

Incidence of oral myiasis is comparatively lesser than that of cutaneous myiasis as oral tissues are not permanently exposed to the external environment [5]. Cases of oral myiasis have been reported to occur following dental extraction, nosocomial infection, in drug addicts, in psychiatric patients, and in conditions that are likely to cause prolonged mouth opening such as mouth breathing during sleep, senility, alcoholism, and mental retardation [4]. Other predisposing factors are incompetent lips, poor oral hygiene, severe halitosis, anterior open bite, facial trauma, extraction wounds, ulcerative lesions, and carcinoma [2, 4, 6]. In this paper, we present a case of oral myiasis caused by* Chrysomya bezziana* involving the anterior maxilla and mucosal surface of upper lip in a 70-year-old female followed by a review of the literature.

## 2. Case Report

**Figure 1:**
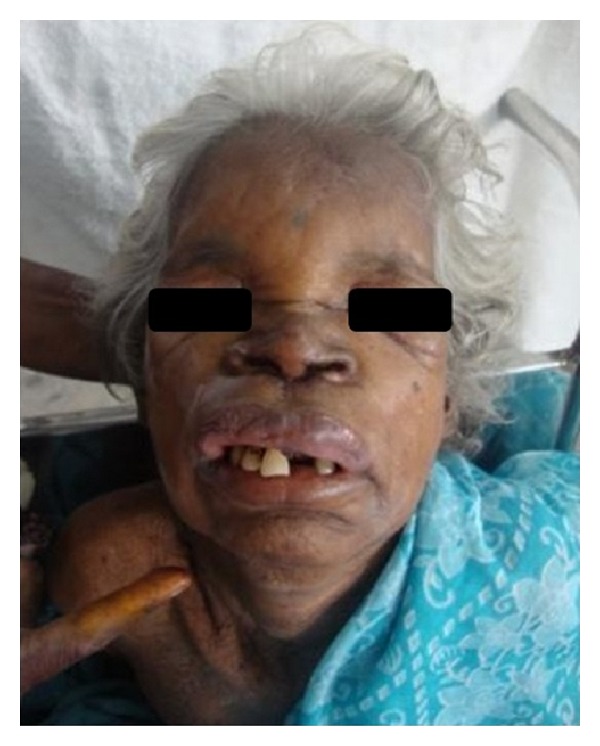
Extraoral photograph revealing diffuse swelling involving the upper lip and the anterior maxilla.

A 70-year-old female presented with a chief complaint of swelling in relation to the upper front teeth since 3 days. She gave history of pain which was of pricking type radiating to the upper half of the face. Extraoral examination revealed a single diffuse swelling measuring 5 × 4 cm^2^ involving the upper lip and the surrounding structures (Figure 1). Skin overlying the swelling appeared normal. On palpation the inspectory findings were confirmed and there was no local rise in temperature and no pulsation; the swelling was firm and tender. There was no numbness or paresthesia in relation to the swelling.

**Figure 2:**
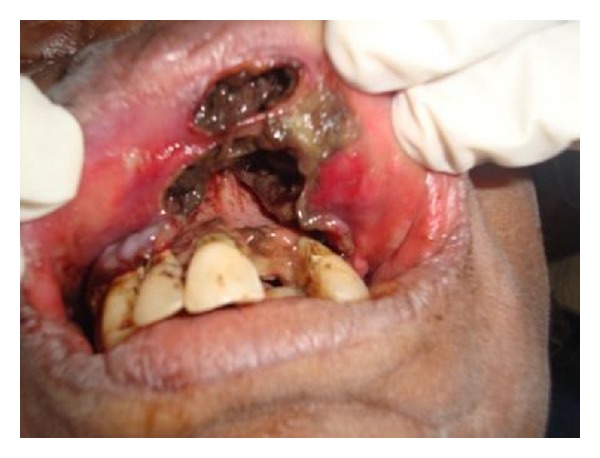
Intraoral photograph revealing necrosis involving labial vestibule and mucosal surface of upper lip.

**Figure 3:**
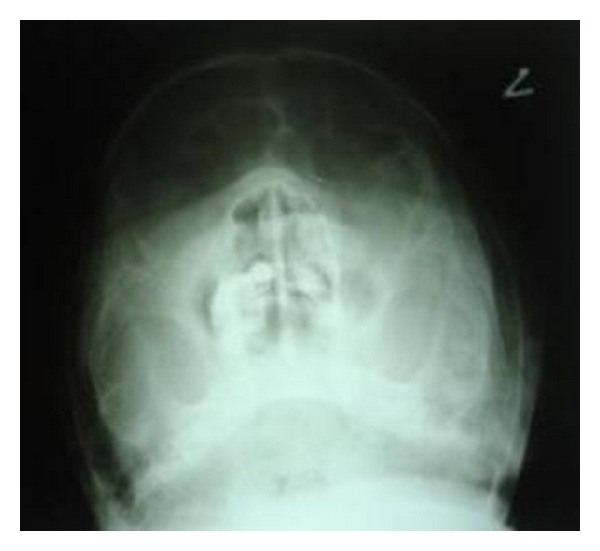
Paranasal sinus view depicting radiolucency involving maxillary anterior region.

Patient was from a low socioeconomic background, was malnourished, and had a poor oral hygiene. Intraoral examination revealed diffuse necrosis of soft tissues in labial vestibule in relation to teeth 11, 12, 13, 14, 21, 22, and 23. The area was soft and tender on palpation. The anterior part of the hard palate showed necrosis and the mucosa covering it was completely detached exposing the underlying bone (Figure 2). A sinus opening was seen present on the mucosal surface of upper lip in relation to tooth 11. Live grayish white maggots were seen crawling through the opening of the sinus. PNS view revealed radiolucency in relation to teeth 11, 12, and 21 suggestive of bone resorption (Figure 3). The hematological report was normal.

**Figure 4:**
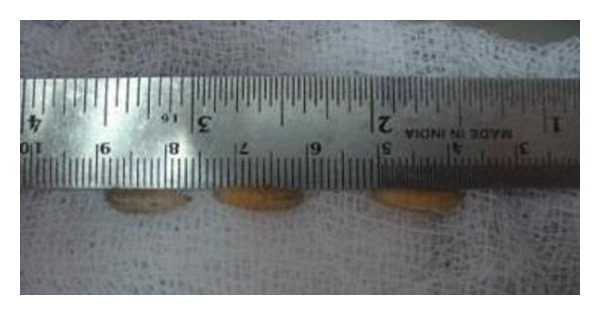
Picture showing live maggots harvested from the affected region.

**Figure 5:**
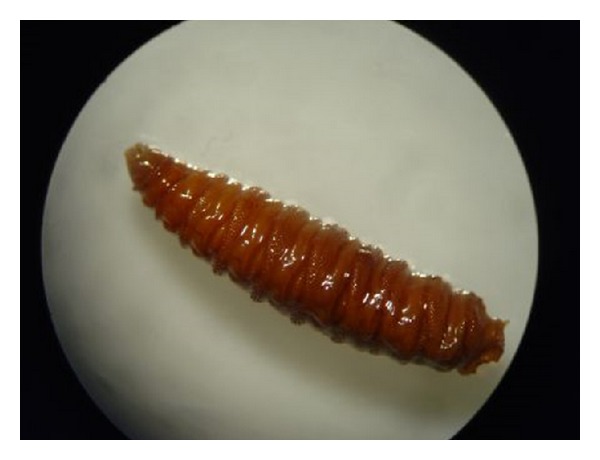
Photomicrograph showing a solitary larva of* Chrysomya bezziana* with tapered body and characteristic transverse grooves.

**Figure 6:**
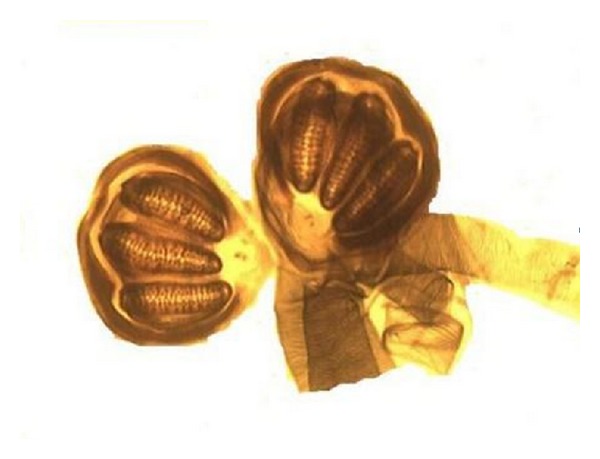
Photomicrograph revealing spiracles typical of* Chrysomya bezziana* on entomological examination.

The patient was treated by removal of the maggots, debridement, and irrigation. The wound was debrided under local anesthesia and roller gauze impregnated with turpentine oil was inserted into the cavity created as a result of tissue necrosis. 30–40 live maggots were harvested from the affected region (Figure 4). Copious irrigation with normal saline and povidine iodine was performed. Under antibiotic coverage with oral penicillin, the patient underwent debridement again until the maggots were completely removed. On entomological examination the maggots were found to be of species* Chrysomya bezziana* (Figures 5 and 6).

## 3. Discussion

Myiasis has been defined by Zumpt as “the infestation of live human and vertebrate animals with dipterous larvae which at least for a certain period feed on the host's dead or living tissue, liquid body substance or ingested food” [7]. Myiasis occurs more commonly in rural area than in urban and predisposing factors may be medical conditions like diabetes mellitus, psychiatric illness, leprosy, mental retardation, patients with an open wound, and those who are mouth-breathers, drunkards, senile, or the hemiplegic [7, 8]. Other risk factors may be poor oral hygiene, facial trauma, suppurative lesions [7]. Myiasis has also been described following teeth extraction [7]. Furthermore, cases with myiasis involving cancerous wounds, such as squamous cell carcinoma, have also been described [9]. In the present case, the patient was from a low socioeconomic background residing in a rural area and had a poor oral hygiene which may be thought of as the predisposing and contributing factors to myiasis in this case.

Based on substrate, myiasis is classified as (a) primary myiasis, when larvae feed on living tissue, and (b) secondary myiasis, when larvae feed on dead tissue. Depending upon the mode of infestation it is of three types: (a) accidental myiasis, when larvae get ingested along with food, (b) semispecific myiasis, when the larvae are laid on necrotic tissue of the wound, and (c) obligatory, when larvae affect undamaged skin. Based on the degree of host dependence, it is classified as (a) obligatory, where fly larvae are completely parasitic and depend upon the host for completion of their life cycle, (b) facultative, where the fly larvae are free living and only circumstantially adapt themselves to parasitic dependence to a host. Based on anatomic site, it can be classified as (a) cutaneous myiasis, (b) myiasis of external orifices, and (c) myiasis of internal organs [10, 11].

Flies causing myiasis belong to the order Diptera [4]. The genera commonly reported are Sarcophagidae, Calliphoridae, Oestridae, and Muscidae from the Diptera order [4].* Chrysomya bezziana*, also known as “Old World Screwworm,” is an obligate parasite and belongs to the order Diptera, family Calliphyridae, and suborder Calliphoridae.

The adult fly of* Chrysomya bezziana* is a green or blue-green fly and widely distributed in tropical and subtropical countries of Africa and Asia, including Southeast Asia, India, Saudi Arabia, Indonesia, the Philippines, Papua, New Guinea, and Persian Gulf.

The development of* Chrysomya bezziana* from egg to adult fly can be completed in 18 days under optimal conditions. The adult female fly lays eggs on live mammals and deposits around 150–200 eggs every two days at the site of the wound in body orifices. The eggs hatch after 12–18 hours and the first-stage larvae, white in color, and 1.5 mm in length will emerge from the eggs and then burrow into wound or wet tissues. In about four days, the larvae moult into the second and third stages, 4–18 mm in length. After 5–7 days, the third-stage larvae would leave the wound and fall to the ground to pupate and transformed into adult fly around seven days later [9, 12].

*Chrysomya bezziana* differs from other maggot infestations by its ability to cause tissue invasion even without preexisting necrosis. The larvae of* Chrysomya bezziana* burrow deep into the host's healthy living tissue in a screw-like fashion feeding on living tissue. In our case also the larvae were present deep into the tissue in the anterior maxilla and this may be responsible for the extensive necrosis observed in the present case [13].

The treatment of myiasis comprises of local and systemic measures [2]. Local measures consist of topical application of turpentine oil, mineral oil, chloroform, ethyl chloride, or mercuric chloride followed by manual removal of the larvae and surgical debridement [4]. Systemic treatment includes broad-spectrum antibiotic such as ampicillin and amoxicillin especially when the wound is secondarily infected [2]. In the present patient, debridement with turpentine oil along with copious irrigation with normal saline and povidone iodine followed by mechanical removal of maggots, and antibiotic coverage with oral penicillin was used for the treatment.

## 4. Conclusion

Albeit oral myiasis is an uncommon condition, is generally self-limiting, and in many cases not dangerous to the host, the clinician should be aware of this disease and should take appropriate measures for its prevention. Prevention of myiasis involves control of fly population, general cleanliness of dwelling areas, maintaining good oral and personal hygiene, and provision for basic sanitation and health education. A very intimate care needs to be taken in medically compromised dependent patients as they are unable to maintain their basic oral hygiene.
